# Comparison of prognostic gene expression signatures for breast cancer

**DOI:** 10.1186/1471-2164-9-394

**Published:** 2008-08-21

**Authors:** Benjamin Haibe-Kains, Christine Desmedt, Fanny Piette, Marc Buyse, Fatima Cardoso, Laura van't Veer, Martine Piccart, Gianluca Bontempi, Christos Sotiriou

**Affiliations:** 1 Functional Genomics Unit, Jules Bordet Institute, Université Libre de Bruxelles, Brussels, Belgium; 2 Machine Learning Group, Université Libre de Bruxelles, Brussels, Belgium; 3 International Drug Development Institute (IDDI), Louvain-La-Neuve, Belgium; 4 Netherlands Cancer Institute, Amsterdam, Netherlands; 5 TRANSBIG consortium, Jules Bordet Institute, Brussels, Belgium

## Abstract

**Background:**

During the last years, several groups have identified prognostic gene expression signatures with apparently similar performances. However, signatures were never compared on an independent population of untreated breast cancer patients, where risk assessment was computed using the original algorithms and microarray platforms.

**Results:**

We compared three gene expression signatures, the 70-gene, the 76-gene and the Gene expression Grade Index (GGI) signatures, in terms of predicting distant metastasis free survival (DMFS) for the individual patient. To this end, we used the previously published TRANSBIG independent validation series of node-negative untreated primary breast cancer patients. We observed agreement in prediction for 135 of 198 patients (68%) when considering the three signatures. When comparing the signatures two by two, the agreement in prediction was 71% for the 70- and 76-gene signatures, 76% for the 76-gene signature and the GGI, and 88% for the 70-gene signature and the GGI. The three signatures had similar capabilities of predicting DMFS and added significant prognostic information to that provided by the classical parameters.

**Conclusion:**

Despite the difference in development of these signatures and the limited overlap in gene identity, they showed similar prognostic performance, adding to the growing evidence that these prognostic signatures are of clinical relevance.

## Background

During the last two decades, several clinical and pathological parameters have been used to evaluate the prognosis of breast cancer (BC) patients. Although different guidelines have been developed to assist clinicians in selecting patients who should receive adjuvant therapy, such as the St Gallen consensus criteria [1], the NIH guidelines [2] or Adjuvant! Online [3], it still remains a challenge to distinguish those patients who would really need adjuvant systemic therapy from those who could be spared such a treatment.

With the advent of array-based technology and the sequencing of the human genome, new insights into BC biology and prognosis have emerged. Interestingly, several groups conducted comprehensive genome-wide assessments of gene expression profiling and identified prognostic gene expression signatures [4-6]. To this end, different approaches have been used: 1/ the "top-down" (data-driven) approach and 2/ the "bottom-up" (hypothesis-driven) approach.

Examples of signatures which were developed using the first approach, i.e. by seeking gene expression profiles that are associated or correlated with clinical outcome without any a priori biological assumption, are the 70- and 76-gene signatures developed by the Netherlands Cancer Institute in Amsterdam with Rosetta Informatics-Merck, and the Erasmus MC in Rotterdam together with Veridex, respectively [5,6]. Although these signatures were built using a different microarray platform and had only a small gene overlap, a feature common to both signatures is that they correctly identified the high-risk patients while also identifying a higher number of low-risk patients not needing treatment compared to the clinical guidelines. In order to investigate the enormous potential of these signatures towards better individualization of treatment options in BC therapy, TRANSBIG, a network for translational research established by the Breast International Group (BIG), recently conducted a validation study of the 70-gene and 76-gene signatures which demonstrated the reproducibility and robustness of the 70- and 76-gene signatures [7,8]. This important validation work has led to the implementation of one of the first prospective clinical trials, MINDACT (Microarray In Node-negative Disease may Avoid Chemotherapy Trial) which evaluates the benefit/risk ratio of chemotherapy when the assessment of prognosis based on clinico-pathological features differs from that provided by the 70-gene signature assessed by the MammaPrint™ [9].

An example of deriving a prognostic gene expression signature using a hypothesis-driven approach was the study reported by our group that focused on histological grade, a well-established pathological parameter rooted in the cell biology of BC [4]. Indeed, clinicians face a huge problem with respect to patients who have intermediate-grade tumors (grade 2), as these tumors, which represent 30% to 60% of cases, are the major source of inter-observer discrepancy and may display intermediate phenotype and survival, making treatment decisions for these patients a great challenge, with subsequent under- or over-treatment. Performing a supervised analysis, we developed a Gene expression Grade Index (GGI) score based on 97 genes. These genes were mainly involved in cell cycle regulation and proliferation and were consistently differentially expressed between low and high grade breast carcinomas. Interestingly, the GGI was able to reclassify patients with histological grade 2 tumors into two groups with distinct clinical outcomes similar to those of histological grade 1 and 3, respectively.

In addition to the signatures described above, many other research groups have contributed gene expression signatures that are predictive of clinical outcome in BC [10]. However, given the fact that their performances were evaluated on different datasets with limited or no independent validation and that there is only little gene overlap between the different gene sets, which can be attributed to the different platforms, training sets, and/or statistical tools used, it is unclear which is the best. The public availability of the TRANSBIG series gives the opportunity to perform a thorough comparison of several gene signatures. Indeed, this dataset of untreated primary BC patients is the only one on which three gene signatures, the 70-gene, the 76-gene and the GGI signatures, were computed using the original algorithms and microarray platforms [7,8], providing also the advantage that this population was not used for the development of any of these signatures. Here, we statistically compared these three signatures in terms of predicting clinical outcome for the individual patient using two performance criteria.

## Methods

### Gene expression and clinical data

Gene expression and clinical data of TRANSBIG series [7,8] were retrieved from EMBL-EBI ArrayExpress (, accession number E-TABM-77) and NCBI GEO (, accession number GSE7390) databases, for the validation of the 70-gene signature (TBAGD) and of the 76-gene signature (TBVDX), respectively. The original TRANSBIG series included 309 patients for whom the 70-gene signature was computed using the Agendia clinical MammaPrint™ 1.9 k Agilent custom microarray chip. This series is referred to as TBAGD. In a second time, the 76-gene signature was computed for a subset of these patients for whom there was enough material left. The Affymetrix HG-U133A research GeneChip™ was used for the signature computation. This series of 198 patients is referred to as TBVDX. Finally, we were able to compute the gene expression grade index in TBVDX as this series used Affymetrix technology. In this paper, we used the TBVDX patient's series for which we had the official classification for the three gene signatures, i.e. the 70-gene, 76-gene and the gene expression grade index.

### Risk status

We considered only the binary risk status for the survival analysis, as the continuous risk scores are not publicly available for 70- and 76-gene signatures in the TBAGD and TBVDX series, respectively. The TBAGD series is composed of 307 BC patients and the TBVDX series is composed of 198 BC patients who are also included in the TBAGD series. In order to use the GGI as a prognostic signature, we first identified a threshold that allows to define the binary risk status according to the GGI scores, on the dataset of 286 patients used by Wang *et al*. (VDX, [6]). Indeed, the threshold used in the original publication [4] was selected to optimize the discrimination between patients with histological grade 1 and 3 tumors. As this threshold was not suited for survival analysis, we used the same training set as the 76-gene signature to keep the TRANSBIG series fully independent. We did not attempt to select a threshold optimizing some performance criteria, e.g. hazard ratio or logrank p-value, in order to avoid overfitting in VDX. Instead, we selected a threshold based on tertiles (the third of the patients having the lowest GGI scores being defined as low-risk and the remaining patients as high-risk) leading to similar repartition of patients in low- and high-risk groups to the 76-gene signature. The GGI score was computed as in Sotiriou *et al*. [4] except that we performed a robust scaling instead of the original scaling method to avoid the use of histological grade information. After the robust scaling, the GGI scores have an interquartile range equals 1 and a median equals 0 within the dataset. The risk status computed using the threshold based on tertiles, yielded good classification performance on the VDX dataset (HR of 2.12; 95% CI: 1.35–3.34; p = 0.001). The GGI continuous risk scores for TBVDX were computed as for VDX and the GGI binary risk status was defined using the threshold identified on VDX. The clinical risk status was defined using the Adjuvant! Online software (AOL, ) as in the validation study conducted by the TRANSBIG consortium [7,8].

### Classification association

We used Cramer's V statistic [11] to quantify the strength of the association between two gene signature classifications. The values range from 0 to 1, with 0 indicating no association and 1 indicating a perfect association. Traditionally, values of 0.36 to 0.49 indicate a substantial association, and values of 0.50 or more indicate a strong association. The significance of such an association was computed using a chi-squared test.

### Survival analysis

We considered the distant metastasis free survival (DMFS), time to distant metastasis (TDM) and overall survival (OS) of BC patients as the survival endpoints. We performed the survival analyses by censoring the survival data at 10 years and by considering the full follow-up. We show the results for DMFS censored at 10 years in the article. The results for TDM and OS censored at 10 years are reported in [Additional File 1]. The survival analyses with the full follow-up are also reported in [Additional File 1]. Sensitivity and specificity were estimated at 3, 5 (the endpoint used to derive the 70- and 76-gene signatures), 10 and 15 years and by considering the full follow-up as well. Sensitivity was defined as the probability that a patient who experienced the event of interest was in the high-risk group and specificity as the probability that a patient who did not experience the event of interest was in the low-risk group. We used the nearest neighbors estimator defined in [12] in order to take into account the time of events and the censoring. Hazard ratios between two groups were estimated through univariate Cox's proportional hazard regression models, stratified by clinical center to account for the possible heterogeneity in patient selection or other potential confounders among the various centers. Hazard ratios for the risk groups defined by the gene signature were also estimated with stratification for clinical risk in order to reflect the prognostic impact of the gene signature over and above that of clinicopathological variables ("adjusted hazard ratios"), as reported previously in [7,8]. In addition to the hazard ratio, we used the concordance index to quantify the predictive ability of a survival model [13]. It estimates the probability that, of a pair of randomly chosen comparable patients, the patient in the high-risk group will recur before the patient in the low-risk group. A pair of patients is comparable if one of the patients recurred before the other patient and if the patients are in different risk groups. Standard error for the concordance index was estimated based on the asymptotic normality of its estimate [14]. The difference in hazard ratios and concordance indices were computed using a paired Student t test. Survival curves were computed through the Kaplan-Meier product limit estimator and their difference was tested in a univariate Cox model, stratified when required.

All p-values were two-tailed and p-values < 0.05 were considered statistically significant. All statistical analyses were carried out using R version 2.5.1 [15].

## Results

### Risk status computed by the prognostic signatures

We used the original algorithms and microarray platforms to compute the risk status of 198 patients used in the second TRANSBIG validation study [8]. Similarly to the GENE70 [5] and GENE76 [6] signatures, we performed a calibration step in order to compute GGI classification in this independent series. This step makes the prediction of a single patient challenging, as it requires a large number of samples. However, the standardization of hybridization protocols, the setup of a central laboratory to carry out the microarray experiments and the use of test samples to calibrate the process might help to avoid this issue. The MAQC consortium [16] is specifically studying this problem in order to bring the microarray-based gene signatures into clinic.

### Patient characteristics according to the prognostic signatures

The patients were younger than the age of 61 (median age 47), had node-negative, T1–T2 (≤ 5 cm) tumors and did not receive any adjuvant treatment. The tumor samples from these patients were previously hybridized on the Agilent platform to define the 70-gene signature [6,17], as well as on the Affymetrix platform, from which the 76-gene signature [6] and Gene expression Grade Index (GGI, [4]) were computed. Patient characteristics are shown in Table 1, organized according to their genomic risk of recurrence as defined by the 70-gene and the 76-gene signatures as well as by the GGI, and by their clinical risk as defined by Adjuvant! Online (AOL, [3,7,8]). The distribution of the risk categories was similar for the different signatures in terms of patient's age and tumor size. However, differences in risk distribution were observed between the 76-gene signature and the two others in terms of tumor grade and estrogen receptor (ER) status. Indeed, the 76-gene signature identified a higher proportion of high-risk grade 2 tumors and low-risk grade 3 tumors, high-risk ER-positive and low-risk ER-negative tumors. When looking at the distribution of the high and low-risk patients according to the ER status, it appears clearly that these signatures mainly impact on the prediction of clinical outcome on ER-positive patients. Compared to the different genomic risk classifications, the clinical risk classification (AOL) identifies a higher proportion of high-risk patients in the older subgroup or in the group of patients with large tumors. None of the patients whose tumors were moderately/poorly differentiated or ER-negative are considered as low-risk by AOL.

**Table 1:** Characteristics of patients of the TRANSBIG validation series (n = 198), according to the 70-gene signature (GENE70), the 76-gene signature (GENE76), the Gene expression Grade Index (GGI) and the Adjuvant! Online (AOL) risk classifications.

Signature	GENE70		GENE76		GGI		AOL	
Number of patients	Low-risk (N = 66)	High-risk (N = 132)	Low-risk (N = 55)	High-risk (N = 143)	Low-risk (N = 69)	High-risk (N = 129)	Low-risk (N = 46)	High-risk (N = 152)

Age								
< 41 years	11	31	10	32	10	32	4	38
41–50 years	33	67	24	76	33	67	33	67
51–60 years	33	34	24	35	26	30	9	47
Size								
T1ab (< 1 cm)	4	5	4	5	4	5	8	1
T1c (1–2 cm)	24	35	17	42	25	34	21	38
T2 (2–5 cm)	38	92	34	96	40	90	17	113
Tumor grade								
Good differentiation	17	13	14	16	20	10	23	7
Intermediate	42	41	21	62	44	39	23	60
Poor differentiation	7	76	20	63	5	78	0	83
Unknown	0	2	0	2	0	2	0	2
Estrogen receptors								
Positive	63	71	41	93	65	69	46	88
Negative	3	61	14	50	4	60	0	64

### Concordance of classification of samples

**Figure 1:**
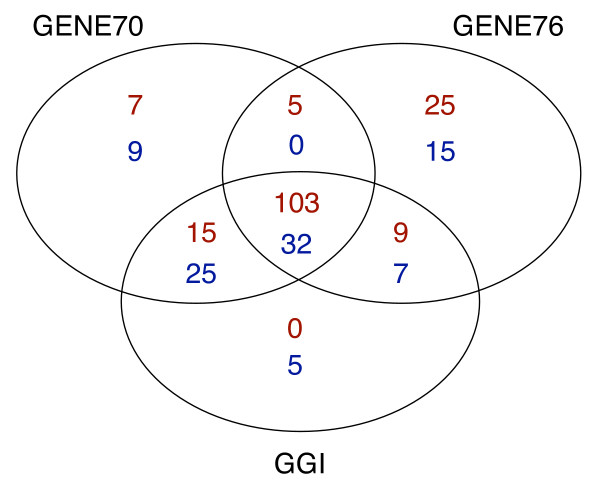
**Venn diagram illustrating the classification of the tumor sample according to the prognostic signatures.** Dark red = high-risk patients and blue = low-risk patients. GENE70 = 70-gene signature, GENE76 = 76-gene signature, and GGI = Gene expression Grade Index.

Figure 1 illustrates the classification of the tumor samples according to the prognostic signatures. We observed agreement in prediction in 135 of 198 patients (68%) when considering the three signatures. When comparing the signatures two by two, agreement in prediction was 71% for the 70- and 76-gene signatures, 76% for the 76-gene signature and the GGI, and 88% for the 70-gene signature and the GGI. The strength of the concordance of classifications, quantified through Cramer's V statistic, was 0.33 for the 70- and 76-gene signatures, 0.47 for the 76-gene signature and the GGI, and 0.76 for the 70-gene signature and the GGI.

### Survival analyses

In this section, we report the results from the survival analyses using the DMFS censored at 10 years and with the full follow-up. We performed these two separate analyses in order to highlight the time-dependency of the gene signatures as shown in [7,8].

#### Survival data censored at 10 years

**Figure 2:**
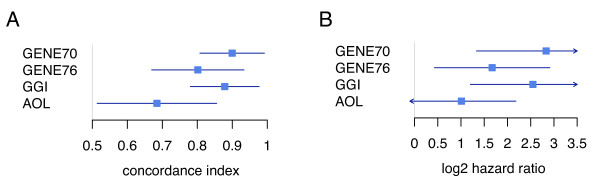
**Forest plots (and 95% CI) for the three gene signatures and the Adjuvant! Online classification showing: A/the concordance indices, and B/the log2 hazard ratios.** GENE70 = 70-gene signature, GENE76 = 76-gene signature, GGI = Gene expression Grade Index and AOL = Adjuvant! Online.

To assess the prognostic ability of the three signatures, we first compared their concordance index, which is used to quantify the predictive ability of a survival model. Although all three concordance indices were highly significant, the 70-gene signature and GGI displayed a higher concordance index compared to the 76-gene signature (0.90 compared to 0.80; Figure 2A). However, this difference was not statistically significant (Table 2). In contrast, the clinical risk calculated using AOL displayed a lower concordance index value (0.69) compared to either ones generated by the genomic signatures.

**Table 2:** P-values of the Student t test for the difference between concordance indices and hazard ratios for the 70-gene signature (GENE70), the 76-gene signature (GENE76), and the Gene expression Grade Index (GGI) risk classifications.

	p-value for difference in concordance indices	p-value for difference in hazard ratios
GENE70 vs GENE76	0.15	0.11
GENE70 vs GGI	0.53	0.42
GENE76 vs GGI	0.22	0.19

**Figure 3:**
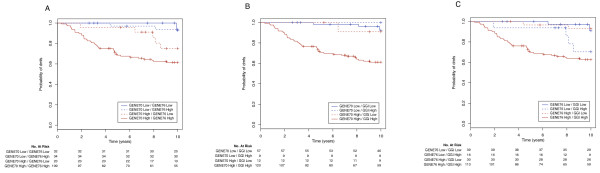
**Kaplan-Meier curves for distant metastasis free survival for: A/the 70-gene signature vs the 76-gene signature; B/the 70-gene signature vs the Gene expression Grade Index, and C/the 76-gene signature vs the Gene expression Grade Index.** GENE70 = 70-gene signature, GENE76 = 76-gene signature and GGI = Gene expression Grade Index.

We next performed univariate and multivariate Cox analyses, which included the traditional clinico-pathological parameters, for each signature separately. The univariate hazard ratios (HR) were 7.12 (95% CI: 2.52–20.11; p = 2.1 × 10^-4^), 3.18 (95% CI: 1.35–7.53; p = 8.4 – 10^-3^) and 5.85 (95% CI: 2.3–15; p = 2.1 – 10^-4^) for the 70-gene signature, 76-gene signature and the GGI respectively. We additionally computed the HR for the clinical risk as defined by AOL, which was not statistically significant for DMFS evaluation in this cohort of patients (2.01; 95% CI: 0.89–4.5; p = 0.091). The log2 of these HR are illustrated in Figure 2B. Although the HR of the 70-gene signature and the GGI were higher than the HR of the 76-gene signature, the differences were not statistically significant (see Table 2). Figure 3 illustrates the Kaplan-Meier estimates of DMFS for the four groups of patients (two groups with concordant results in risk assessment and two with discordant results) for the different signatures two by two.

From the multivariate analyses (Table 3), we can conclude that the three signatures added significant information to the traditional parameters and were the strongest predictive variables of DMFS, as reflected by their lowest p-values compared to the other variables. The additional information of these signatures over the clinical risk was also confirmed by the fact that the univariate HRs for the three signatures remained similar when adjusted for the clinical risk, with a HR of 7.25 (95% CI: 2.4–21.5; p = 3.5 – 10^-4^), 2.8 (95% CI: 1.2–6.8; p = 0.018) and 6.25 (95% CI: 2.3–17; p = 3.3 – 10^-4^) for the 70-gene signature, 76-gene signature and the GGI respectively.

**Table 3:** Multivariate Cox analyses for the 70-gene signature (GENE70), the 76-gene signature (GENE76) and the Gene expression Grade Index (GGI) risk classifications.

	GENE70		GENE76		GGI	
	
	HR (95% CI)	p-value	HR (95% CI)	p-value	HR (95% CI)	p-value
Age (≤ or >50 years)	1.51 (0.82–2.79)	0.3	1.78 (0.97–3.25)	0.062	1.73 (0.94–3.16)	0.077
Tumor Size (≤ or >2 cm)	1.3 (0.72–2.5)	0.36	1.27 (0.68–2.37)	0.45	1.22 (0.65–2.29)	0.53
ER status	0.82 (0.43–1.6)	0.57	0.6 (0.31–1.17)	0.13	0.78 (0.4–1.51)	0.46
Grade	0.93 (0.34–2.53)	0.89	1.51 (0.53–4.28)	0.5	0.75 (0.27–2.08)	0.58
Risk according to the gene signature	7.1 (2.4–21)	4 × 10^-4^	3.39 (1.41–8.12)	6 × 10^-3^	6.42 (2.36–17.45)	3 × 10^-4^

Lastly, we combined the three gene signatures in order to assess the potential improvement in BC prognostication. We used a simple combination scheme that defined the risk of a patient as the sum of the classifications (low-risk = 0 and high-risk = 1) by the three gene signatures. As illustrated in Supplementary Figure 1 in [Additional File 1], the patients for whom the three gene signature classifications were concordant are well defined, with only 2 patients relapsing in the low-risk group after 9 years of follow up. However, the patients with discordant classifications exhibited good survival and their survival curves were not distinguishable. The combination of the three gene signatures did not yield significant improvement in prognostication (the hazard ratio between the concordant cases, i.e. 'All Low' and 'All High', is not significantly higher than when each gene signature was considered separately), maybe due to their high concordance and the sample size of the TBVDX series

#### Survival data with the full follow-up

We computed the concordance index of all the gene signatures using the survival data with the full follow-up. The three concordance indices were significant. We observed higher concordance indices for the 70-gene signature and GGI compared to the 76-gene signature (0.84 and 0.79 for GENE70 and GGI respectively compared to 0.71 for GENE76; Supplementary Figure 12 in [Additional File 1]). This difference was not statistically significant (Supplementary Table 5 in [Additional File 1]) although we noted a trend for GENE70 to have a higher concordance index (p = 0.065). In contrast, the clinical risk calculated using AOL displayed a lower concordance index value (0.69) compared to either ones generated by the genomic signatures.

We next performed univariate and multivariate Cox analyses, which included the traditional clinico-pathological parameters, for each signature separately. The univariate hazard ratios (HR) were 2.77 (95% CI: 1.41–5.43; p = 3.1 – 10^-3^), 1.76 (95% CI: 0.92–3.34; p = 0.086) and 2.41 (95% CI: 1.29–4.5; p = 5.9 – 10^-3^) for the 70-gene signature, 76-gene signature and the GGI respectively. We additionally computed the HR for the clinical risk as defined by AOL, which was not statistically significant for DMFS evaluation in this cohort of patients (1.5; 95% CI: 1.29–4.5; p = 0.11). The log2 of these HR are illustrated in Supplementary Figure 13 in [Additional File 1]. Although the HR of the 70-gene signature and the GGI were higher than the HR of the 76-gene signature, the differences were not statistically significant (see Supplementary Table 5 in [Additional File 1]). Supplementary Figures 14–16 in [Additional File 1] illustrate the Kaplan-Meier estimates of DMFS for the four groups of patients for the different signatures two by two.

From the multivariate analyses (Supplementary Table 6 in [Additional File 1]), we can conclude that the three signatures added significant information to the traditional parameters and were the strongest predictive variables of DMFS, as reflected by their lowest p-values compared to the other variables. We computed the univariate HRs adjusted for the clinical risk, i.e. 2.8 (95% CI: 1.35–5.82; p = 5.8 – 10^-3^), 1.55 (95% CI: 0.81–2.97; p = 0.18) and 2.13 (95% CI: 1.12–4.02; p = 0.02) for the 70-gene signature, 76-gene signature and the GGI respectively.

Contrary to the analyses using the survival data censored at 10 years, the HRs with and without adjustment for clinical risk were not significant for GENE76, highlighting the decrease in performance we observed by using the survival data with the full follow-up. This performance degradation was due to a group of late relapses occurring after 10 years of follow-up, classified as low-risk by the three gene signatures (see Supplementary Figure 17 in [Additional File 1]).

We combined the three gene signatures using the method described previously. In agreement with the results from survival data censored at 10 years, the combination did not yield significant improvement in prognostication (see Section 2.1.1 in [Additional File 1]).

#### Sensitivity and specificity

We computed the sensitivity and the specificity for DMFS at 5 years for the three signatures as well as for the clinical risk as defined by AOL. These signatures exhibited high sensitivities (0.97 to 1) compared to the clinical risk (0.88). Similarly to the results reported in previous publications [5-8,18,19], the gene signatures exhibited low specificities (0.33 to 0.42) which were however higher than the specificity associated with clinical risk assessment (0.26). The estimations of sensitivity and specificity for DMFS, TDM and OS at 3, 5, 10, and 15 years and by considering the full follow-up, are given in Supplementary Tables 7, 10 and 13 [see Additional File 1]. Although the gene signatures yielded higher sensitivities and specificities than clinical risk until 10 years, we observed a decrease in performance with increasing follow-up duration (10 years and more). The specificities of the gene signatures remained higher than clinical risk but their sensitivities were slightly lower.

The results were highly concordant between the survival endpoints, namely DMFS, TDM and OS [see Additional File 1].

## Discussion and conclusion

The objective of this study was to conduct an unbiased comparison of three different prognostic signatures. To this end, the signatures had to be evaluated on their original platform and computed with their original algorithms on an independent population of untreated BC patients. All these requirements were met by the TRANSBIG validation series [7,8]. The results showed that the three evaluated signatures had similar capabilities of predicting DMFS (TDM and OS [see Additional File 1]) in this set of patients and added significant prognostic information to that provided by the classical parameters.

Two groups recently undertook to compare different prognostic signatures. Fan *et al*. reported that the intrinsic subtypes and several prognostic signatures [5,6,20-24] gave similar outcome predictions for the individual patient when investigated on a single dataset [25]. Although this study yielded promising conclusions, some issues remained open: 1/ the dataset which was considered for this study was used for the development of some gene sets and could then not be considered as a true independent validation set for all the evaluated signatures and 2/ since some of these signatures were developed on another platform, the initial algorithms could not be applied. Thomassen *et al*. compared nine prognostic signatures in a cohort of low-malignancy BC patients [26]. In their study, they also compared the same signatures as we did, but computed the associated risk classification from data generated on a different platform, with as consequence that not all the genes from the 76-gene signature and the GGI were represented and that they could not use the original algorithms. Although the proportions of samples that they reported with identical classification were slightly higher to ours, the rank of concordances was similar with 83% between the 70- and 76-gene signatures, 85% between the 76-gene signature and the GGI, and 92% (the highest agreement) between the 70-gene signature and the GGI.

Thanks to the long follow-up of the TRANSBIG series (up to 25 years), we were able to assess the performance of the three gene signatures with respect to the time. In agreement with Buyse *et al*. [7] and Desmedt *et al*. [8], we observed a strong time dependence of the classification performance. The gene signatures yielded good performance at 10 years but we observed a strong degradation when considering the full follow-up due to the poor identification of late relapses (after 10 years). That might be due to: (i) the biology, Klein *et al. *have suggested that the biological phenomenon responsible for the appearance of early and late relapses might be different [27,28]; (ii) the statistical method, the GENE70 and GENE76 signatures have been developed to predict early relapses (distant metastasis before 5 years) as in the original publications, the authors controlled the sensitivity and the specificity of these signatures for early relapses only; (iii) the quality of survival data, although it is hard to quantify, one could intuitively think that the quality of survival data decreases with respect to the duration of follow-up since it is difficult to follow patients during a long period (high level of censoring).

While there are only partial or very small to no overlaps between the different prognostic gene signatures [29], there is still a relatively high agreement of classification of the patients between the different signatures. We may assume that these similar outcome predictions are based on representation of largely overlapping biological processes. This is supported by several reports. Indeed, Thomassen *et al*. found that cell cycle and cell proliferation represented the predominant overlaps in gene ontology categories of the nine prognostic signatures they compared [26]. Yu *et al*. also conducted pathway analyses of five published prognostic gene signatures and also found that the signatures had many pathways in common such as cell cycle, regulation of cell cycle, mitosis, apoptosis, etc [29]. Our group also investigated in a large meta-analysis of publicly available gene expression data extensive analysis how different gene lists may give rise to signatures with equivalent prognostic performance and found by dissecting these signatures according to the main molecular processes involved in BC, that proliferation may be the common driving force of several prognostic signatures [30]. This might explain why the combination of the three gene signatures evaluated in this study did not yield significant improvement in prognostication.

Until now, the generation of the prognostic signatures has been done on global sets of BC patients. However, since it is clear that BC is a molecular heterogeneous disease, with subgroups defined primarily by the estrogen (ER) and HER2 receptors, prognosis could be refined to these molecularly homogeneous subgroups of patients. We showed for example in our meta-analysis that proliferation is the strongest parameter predicting clinical outcome in the ER+/HER2- subgroup of patients only, whereas immune response and tumor invasion appear to be the main biological processes associated with prognosis in the ER-/HER2- and HER2+ subgroups respectively [30]. This could also have implications with regard to the evaluation of response to different therapies [31] and help to define new therapeutic strategies in the specific molecular subgroups of BC patients.

To conclude, our study showed that although prognostic signatures may have been developed using a different approach, different platforms and statistical tools on different sets of comparable patients, with a small overlap in gene identity as a consequence, they can result in similar predictions of outcome. Although the technology used has been shown to be ready for clinical practice [16], and can be used as one parameter in combination with current clinical parameters, these signatures need to be prospectively validated to prove their superiority and benefit above and beyond the use of standard clinico-pathological prognosis variables to guide the choice of adjuvant therapy. Two gene signatures, the 70-gene signature which has been studied in this paper, and the recurrence score [21] have reached the final step of prospective testing in the MINDACT (Microarray in Node Negative Disease May Avoid Chemotherapy) and TAILORx trials, respectively. We believe that the results from these studies will help to guide future BC treatment.

## Competing interests

Christos Sotiriou, Mauro Delorenzi and Martine Piccart are named inventors on a patent application for the Genomic Grade signature used in this study. Laura van't Veer is founder and stock owner of Agendia. There are no other conflicts of interest.

## Authors' contributions

BH–K, CD, CS were responsible for the design and execution of the study, data and statistical analysis and interpretation, and final manuscript writing; FP, MB, GB, FC, LV, MP supported the data and statistical analysis and interpretation; GB, CS supervised the study. MP, CS provided the study funding. All authors read and approved the final manuscript.

## Supplementary Material

Additional file 1 Supplementary information. Click here for file
