# Supplementary material for: Comparison of prognostic gene expression signatures for breast cancer
Source: BMC Genomics. 2008 Aug 21;9:394. doi: 10.1186/1471-2164-9-394 (PMC2533026; doi:10.1186/1471-2164-9-394)
Supplement: Additional file 1 — Supplementary information. [file 1471-2164-9-394-S1.pdf]

# Supplementary Information

# Contents


**1 Survival Data Censored At 10 Years** **3**
1.1 Distant Metastasis Free Survival (DMFS) . . . . . . . . . . . . . . . . . . . . . 3
1.1.1 Combining GENE70, GENE76 and GGI in TBVDX . . . . . . . . . . 3
1.2 Time to Distant Metastasis (TDM) . . . . . . . . . . . . . . . . . . . . . . . . . . 5
1.3 Overall Survival (OS) . . . . . . . . . . . . . . . . . . . . . . . . . . . . . . . . 10

**2 Survival Data with the Full Follow-Up** **15**
2.1 Distant Metastasis Free Survival (DMFS) . . . . . . . . . . . . . . . . . . . . . 15
2.1.1 Combining GENE70, GENE76 and GGI in TBVDX . . . . . . . . . . 19
2.2 Time to Distant Metastasis (TDM) . . . . . . . . . . . . . . . . . . . . . . . . . . 20
2.3 Overall Survival (OS) . . . . . . . . . . . . . . . . . . . . . . . . . . . . . . . . 24

# 1 Survival Data Censored At 10 Years

The sections below report the results from the survival analysis considering the distant metastasis free survival (DMFS), time to distant metastasis (TDM) and the overall survival (OS) censored at 10 years as survival endpoint.

## 1.1 Distant Metastasis Free Survival (DMFS)

### 1.1.1 Combining GENE70, GENE76 and GGI in TBVDX

In order to assess the potential improvement in prognostication by combining the classifications computed from the three gene signatures, we applied the following method:

- Each gene classifier is considered as an expert who classifies the patients in low-risk group (vote 0) or high-risk group (vote 1).
- The final classification is the sum of the votes given by each gene classifier.
- In our case we have four different categories:
  1. All the gene classifiers identified the patient as low risk. This class is referred to as '**All Low**'.
  2. Two gene classifiers identified the patient as low risk but one at high risk. This class is referred to as '**2 Low / 1 High**'.
  3. A single gene classifier identified the patient as low risk but the other two as high risk. This class is referred to as '**1 Low / 2 High**'.
  4. All the gene classifiers identified the patient as high risk. This class is referred to as '**All High**'.

The Supplementary Figure 1 sketches the survival curves for each category.

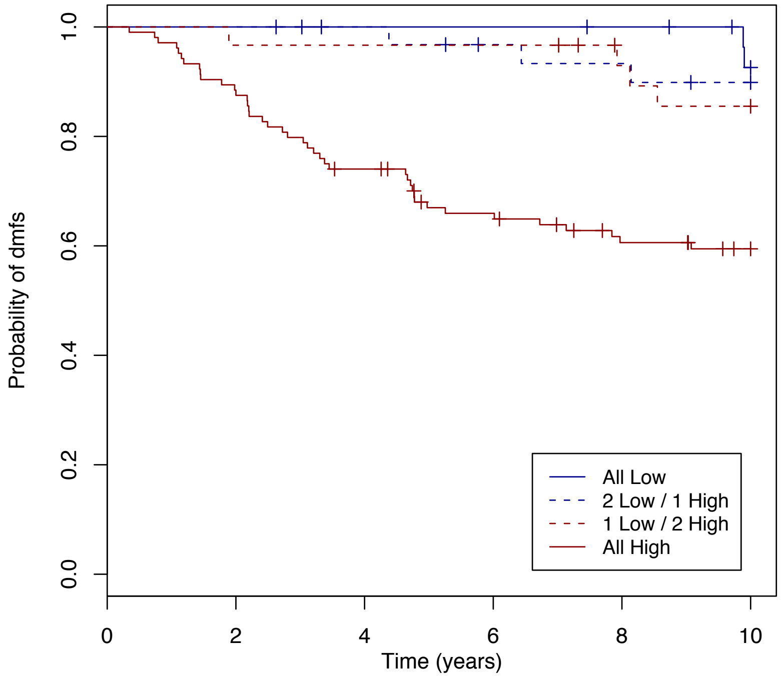


**No. At Risk**

| | 0 | 2 | 4 | 6 | 8 | 10 |
|---|---|---|---|---|---|---|
| All Low | 32 | 32 | 31 | 31 | 30 | 25 |
| 2 Low / 1 High | 32 | 32 | 32 | 29 | 28 | 25 |
| 1 Low / 2 High | 30 | 30 | 30 | 30 | 26 | 23 |
| All High | 104 | 92 | 77 | 65 | 56 | 50 |

Supplementary Figure 1: Combination of the three gene signature classifications.

## 1.2 Time to Distant Metastasis (TDM)

To compare the prognostic ability of the three signatures considering TDM as survival endpoint, we first compared their concordance index, which is used to quantify the predictive ability of a survival model. Although the three indices were highly significant, the 70-gene and genomic grade signatures displayed a higher concordance index compared to the 76-gene signature (0.96 compared to 0.91; Supplementary Figure 2). However this difference was not statistically significant (Supplementary Table 1). Opposed to these high indices, the clinical risk calculated using Adjuvant! Online only displayed a concordance index of 0.75.

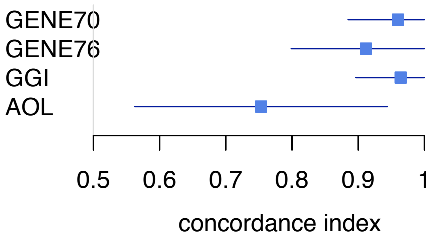


Supplementary Figure 2: Forest plots (and 95% CI) for the three gene signatures and the Adjuvant! Online classification showing the concordance indices considering TDM as survival endpoint.

| | p-value for difference in concordance indices | p-value for difference in hazard ratios |
|---|---|---|
| GENE70 vs GGI | 0.88 | 0.99 |
| GENE76 vs GENE70 | 0.43 | 0.33 |
| GENE76 vs GGI | 0.3 | 0.26 |

Supplementary Table 1: P-values of the Student t-test for the difference between concordance indices and hazard ratios considering TDM as survival endpoint.

We next performed univariate and multivariate Cox analyses, which included the traditional clinico-pathological parameters, for each signature separately. The univariate hazard ratios (HR) were 21.5 (95% CI: 2.84-162.9, p= 3 x $10^{-3}$), 8.44 (95% CI: 2.03-35.13; p= 3.4 x $10^{-3}$) and 23.48 (95% CI: 3.21-172, p= 1.9 x $10^{-3}$) for the 70-gene signature, 76-gene signature and the GGI respectively. We additionally computed the HR for the clinical risk as defined by Adjuvant! Online that is equal to 2.86; 95% CI: 1.01- 8.12; p= 4.9 x $10^{-2}$). The $\log_2$ of these HR are illustrated in Supplementary Figure 3. Although the HR of the 70-gene signature and the GGI were higher than the HR of the 76-gene signature, the differences were not statistically significant (see Supplementary Table 1).

Supplementary Figures 5, 4 and 6 illustrate the Kaplan-Meier estimates of TDM for the four groups of patients (two groups with concordant results in risk assessment and two with discordant results) for the different signatures two by two.

From the multivariate analyses (Supplementary Table 2), we can conclude that the three signatures added significant information to the traditional parameters and were the strongest predictive variables of TDM, as reflected by their lowest p-values compared to the other variables. The additional information of these signatures over the clinical risk was also confirmed

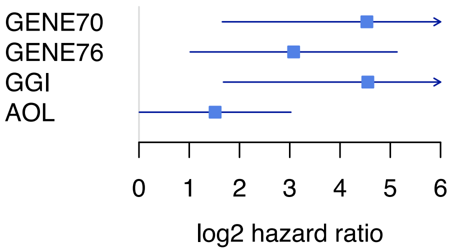


Supplementary Figure 3: Forest plots (and 95% CI) for the three gene signatures and the Adjuvant! Online classification showing the $\log_2$ hazard ratios considering TDM as survival endpoint.

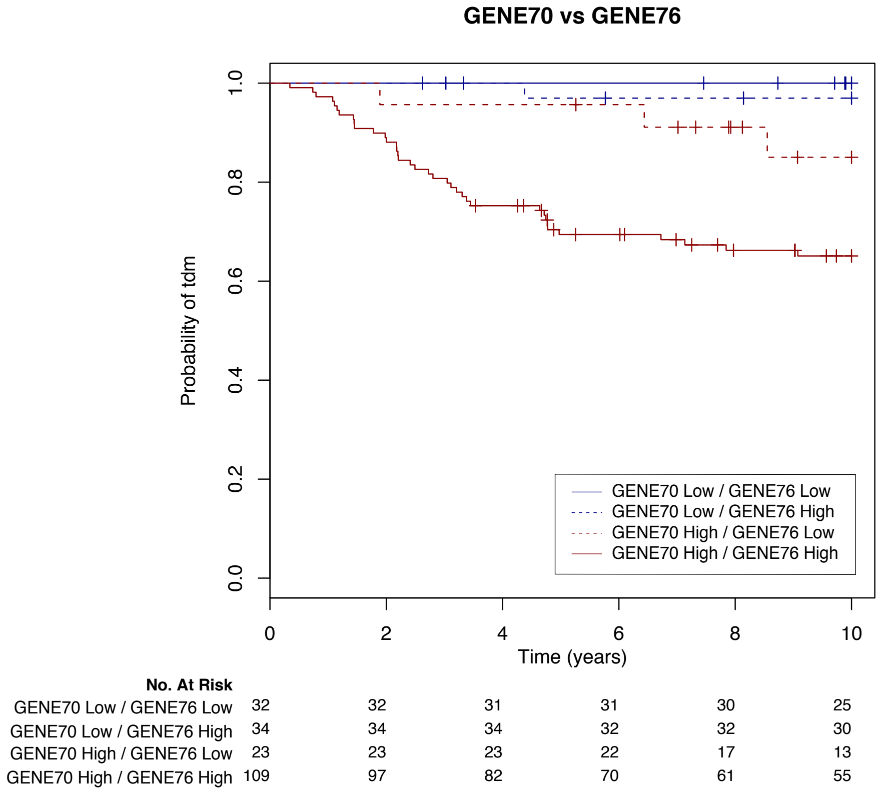


Supplementary Figure 4: Kaplan-Meier curves for time to distant metastasis for the 70-gene signature vs the 76-gene signature.

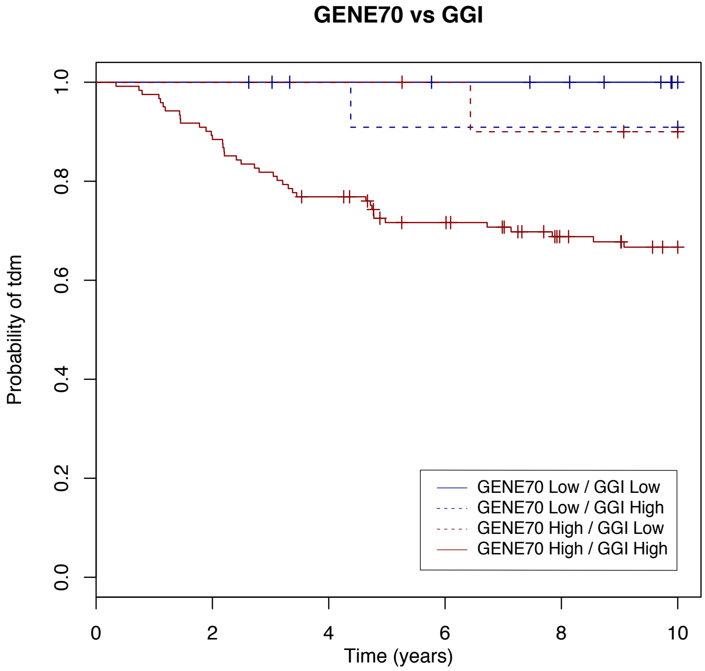


| **No. At Risk** | | | | | | |
|---|---|---|---|---|---|---|
| GENE70 Low / GGI Low | 55 | 55 | 53 | 52 | 51 | 45 |
| GENE70 Low / GGI High | 11 | 11 | 11 | 11 | 11 | 10 |
| GENE70 High / GGI Low | 11 | 11 | 11 | 11 | 10 | 8 |
| GENE70 High / GGI High | 121 | 108 | 93 | 81 | 68 | 60 |

Supplementary Figure 5: Kaplan-Meier curves for time to distant metastasis for the 70-gene signature vs the genomic grade signature.

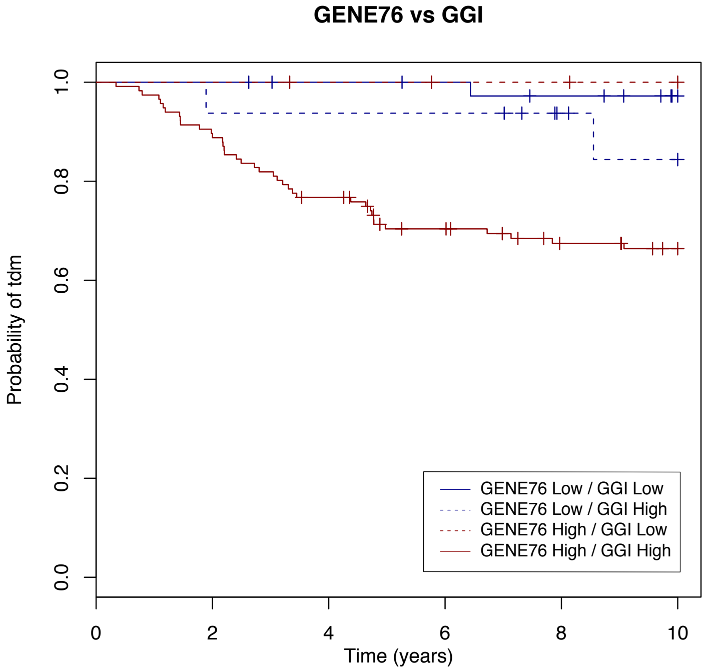


| No. At Risk | | | | | | |
|---|---|---|---|---|---|---|
| GENE76 Low / GGI Low | 39 | 39 | 38 | 37 | 35 | 29 |
| GENE76 Low / GGI High | 16 | 16 | 16 | 16 | 12 | 9 |
| GENE76 High / GGI Low | 27 | 27 | 27 | 26 | 26 | 24 |
| GENE76 High / GGI High | 116 | 104 | 89 | 76 | 67 | 61 |

Supplementary Figure 6: Kaplan-Meier curves for time to distant metastasis for the 76-gene signature vs the genomic grade signature.

by the fact that the univariate HRs for the three signatures remained similar when adjusted for the clinical risk, with a HR of 21.96 (95% CI: 2.92-165.4, p=2.7 x $10^{-4}$), 7.53 (95% CI: 1.8-31.42, p=5.6 x $10^{-3}$) and 20.33 (95% CI: 2.75-150.3, p=3.2 x $10^{-3}$) for the 70-gene signature, 76-gene signature and the genomic grade signature respectively.

| | GENE70 | | GENE76 | | GGI | |
|---|---|---|---|---|---|---|
| | HR (95% CI) | p-value | HR (95% CI) | p-value | HR (95% CI) | p-value |
| Age ($\leq$ or $>$ 50years) | 1.43 (0.71-2.85) | 0.31 | 1.8 (0.91-3.53) | 0.091 | 1.67 (0.84-3.3) | 0.14 |
| Tumor size ($\leq$ or $>$ 2cm) | 1.48 (0.74-2.96) | 0.27 | 1.39 (0.7-2.78) | 0.35 | 1.3 (0.64-2.62) | 0.47 |
| ER status | 0.97 (0.48-2) | 0.93 | 0.69 (0.34-1.43) | 0.32 | 0.92 (0.46-1.87) | 0.82 |
| Grade | 1.37 (0.44-4.23) | 0.59 | 2.54 (0.76-8.48) | 0.13 | 1.11 (0.35-3.56) | 0.86 |
| Risk according to the gene signature | 21.5 (2.84-162.89) | 3 x $10^{-3}$ | 8.66 (2.01-36.43) | 3 x $10^{-3}$ | 23.18 (3.04-177) | 2 x $10^{-3}$ |

Supplementary Table 2: Multivariate Cox analyses for the 70-gene signature (GENE70), the 76-gene signature (GENE76) and the genomic grade (GGI) risk classifications considering TDM as survival endpoint.

## 1.3 Overall Survival (OS)

To compare the prognostic ability of the three signatures considering OS as survival endpoint, we first compared their concordance index, which is used to quantify the predictive ability of a survival model. Although the three indices were highly significant, the 70-gene and genomic grade signatures displayed a higher concordance index compared to the 76-gene signature (0.88, 0.91, and 0.84 for the 70-, 76-gene and genomic grade signatures respectively; Supplementary Figure 7). However this difference was not statistically significant (Supplementary Table 3). Opposed to these high indices, the clinical risk calculated using Adjuvant! Online only displayed a concordance index of 0.65.

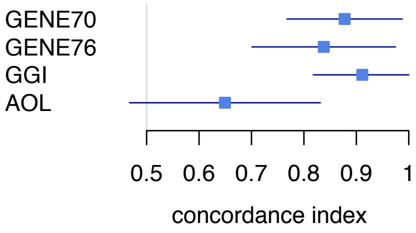


Supplementary Figure 7: Forest plots (and 95% CI) for the three gene signatures and the Adjuvant! Online classification showing the concordance indices considering OS as survival endpoint.

| | p-value for difference in concordance indices | p-value for difference in hazard ratios |
|---|---|---|
| GENE70 vs GGI | 0.37 | 0.92 |
| GENE76 vs GENE70 | 0.59 | 0.29 |
| GENE76 vs GGI | 0.24 | 0.25 |

Supplementary Table 3: P-values of the Student t-test for the difference between concordance indices and hazard ratios considering OS as survival endpoint.

We next performed univariate and multivariate Cox analyses, which included the traditional clinico-pathological parameters, for each signature separately. The univariate hazard ratios (HR) were 6.28 (95% CI: 2.2-17.87, p= 5.8 x $10^{-4}$), 3.34 (95% CI: 1.31-8.53; p= 1.2 x $10^{-2}$) and 6.04 (95% CI: 2.14-17.07; p= 7 x $10^{-4}$) for the 70-gene signature, 76-gene signature and the GGI respectively. We additionally computed the HR for the clinical risk as defined by Adjuvant! Online was not statistically significant for OS evaluation in this cohort of patients (1.01; 95% CI: 0.78- 4.02; p= 1.7 x $10^{-1}$). The $\log_2$ of these HR are illustrated in Supplementary Figure 8. Although the HR of the 70-gene signature and the GGI were higher than the HR of the 76-gene signature, the differences were not statistically significant (see Supplementary Table 3).

Supplementary Figures 10, 9 and 11 illustrate the Kaplan-Meier estimates of OS for the four groups of patients (two groups with concordant results in risk assessment and two with discordant results) for the different signatures two by two.

From the multivariate analyses (Supplementary Table 4), we can conclude that the three

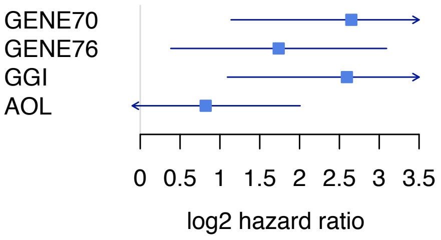


Supplementary Figure 8: Forest plots (and 95% CI) for the three gene signatures and the Adjuvant! Online classification showing the $\log_2$ hazard ratios considering TDM as survival endpoint.

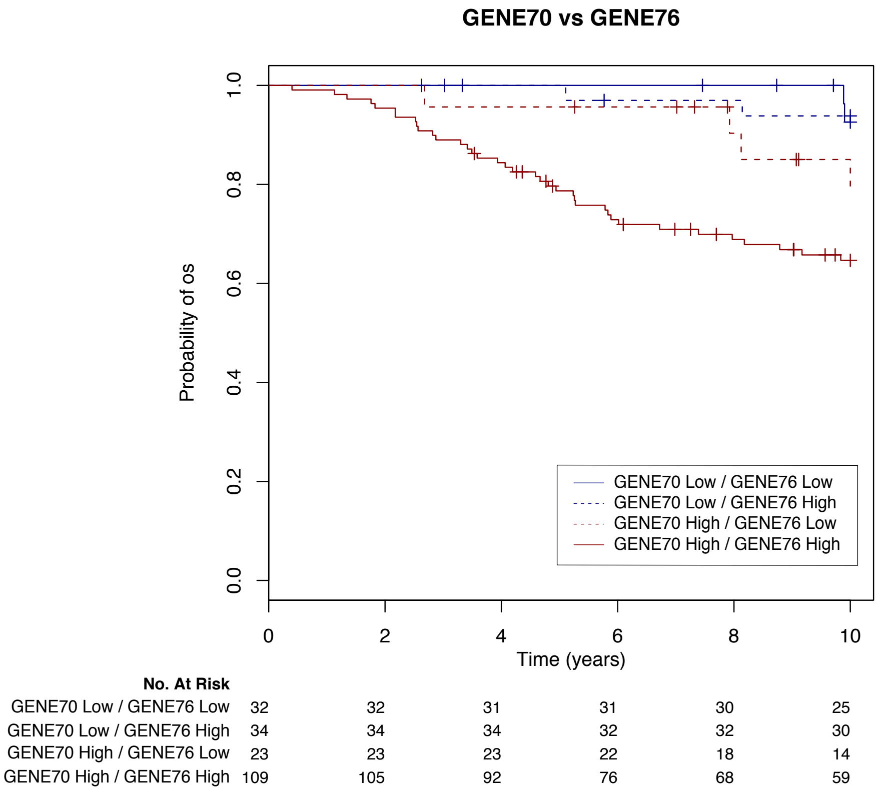


Supplementary Figure 9: Kaplan-Meier curves for overall survival for the 70-gene signature vs the 76-gene signature.

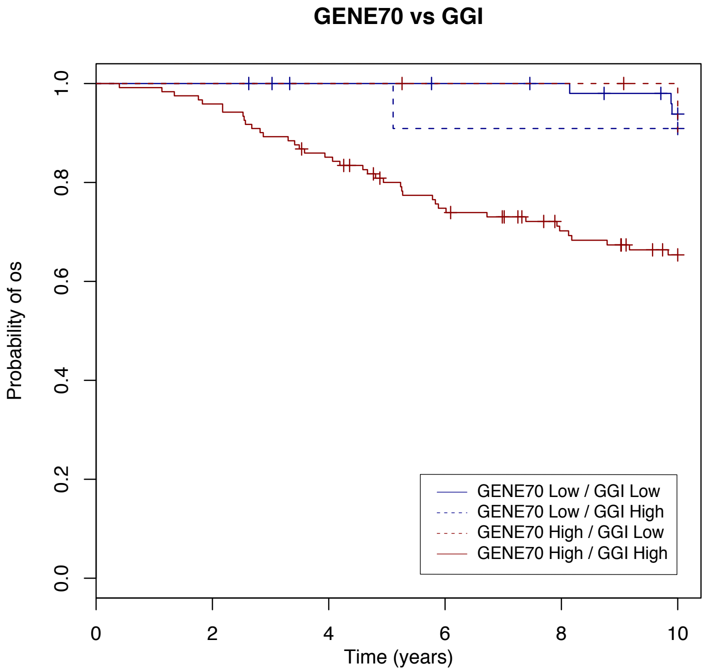


| No. At Risk | 0 | 2 | 4 | 6 | 8 | 10 |
|---|---|---|---|---|---|---|
| GENE70 Low / GGI Low | 55 | 55 | 53 | 52 | 51 | 45 |
| GENE70 Low / GGI High | 11 | 11 | 11 | 11 | 11 | 10 |
| GENE70 High / GGI Low | 11 | 11 | 11 | 11 | 11 | 9 |
| GENE70 High / GGI High | 121 | 117 | 103 | 87 | 75 | 64 |

Supplementary Figure 10: Kaplan-Meier curves for overall survival for the 70-gene signature vs the genomic grade signature.

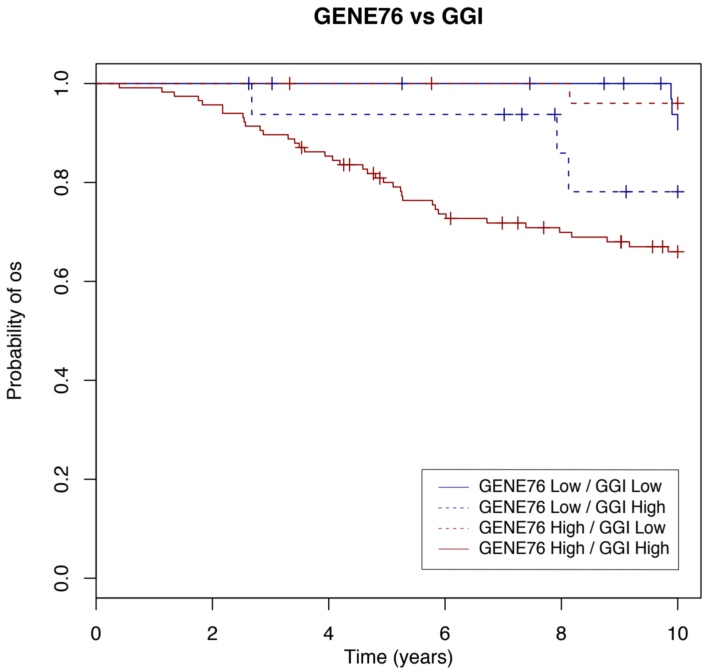


| No. At Risk | | | | | | |
|---|---|---|---|---|---|---|
| GENE76 Low / GGI Low | 39 | 39 | 38 | 37 | 36 | 30 |
| GENE76 Low / GGI High | 16 | 16 | 16 | 16 | 12 | 9 |
| GENE76 High / GGI Low | 27 | 27 | 27 | 26 | 26 | 24 |
| GENE76 High / GGI High | 116 | 112 | 99 | 82 | 74 | 65 |

Supplementary Figure 11: Kaplan-Meier curves for overall survival for the 76-gene signature vs the genomic grade signature.

signatures added significant information to the traditional parameters and were the strongest predictive variables of OS, as reflected by their lowest p-values compared to the other variables. The additional information of these signatures over the clinical risk was also confirmed by the fact that the univariate HRs for the three signatures remained similar when adjusted for the clinical risk, with a HR of 6.46 (95% CI: 2.14-19.44, p=9.2 x $10^{-4}$), 3 (95% CI: 1.17-7.7, p=2.3 x $10^{-2}$) and 5.59 (95% CI: 1.93-16.18, p=1.5 x $10^{-3}$) for the 70-gene signature, 76-gene signature and the genomic grade signature respectively.

| | GENE70 | | GENE76 | | GGI | |
|---|---|---|---|---|---|---|
| | HR (95% CI) | p-value | HR (95% CI) | p-value | HR (95% CI) | p-value |
| Age (≤ or > 50years) | 1.38 (0.72-2.65) | 0.34 | 1.7 (0.89-3.2) | 0.11 | 1.66 (0.86-3.15) | 0.12 |
| Tumor size (≤ or > 2cm) | 1.23 (0.64-2.36) | 0.53 | 1.19 (0.62-2.31) | 0.6 | 1.13 (0.58-2.18) | 0.73 |
| ER status | 0.62 (0.30-1.25) | 0.18 | 0.452 (0.22-0.93) | 0.03 | 0.6 (0.3-1.21) | 0.15 |
| Grade | 0.63 (0.22-1.81) | 0.4 | 0.96 (0.33-2.83) | 0.94 | 0.53 (0.18-1.55) | 0.25 |
| Risk according to the gene signature | 6.27 (2.1-18.8) | 1 x $10^{-3}$ | 3.58 (1.38-9.3) | 8.8 x $10^{-3}$ | 6.9 (2.29-20.81) | 6 x $10^{-4}$ |

Supplementary Table 4: Multivariate Cox analyses for the 70-gene signature (GENE70), the 76-gene signature (GENE76) and the genomic grade (GGI) risk classifications considering OS as survival endpoint.

## 2 Survival Data with the Full Follow-Up

The sections below report the results from the survival analysis considering the distant metastasis free survival (DMFS), the time to distant metastasis (TDM) and the overall survival (OS) with the full followup as survival endpoint.

### 2.1 Distant Metastasis Free Survival (DMFS)

To compare the prognostic ability of the three signatures considering TDM as survival endpoint, we first compared their concordance index, which is used to quantify the predictive ability of a survival model. Although the three indices were highly significant, the 70-gene and genomic grade signatures displayed a higher concordance index compared to the 76-gene signature (0.84 and 0.79 for GENE70 and GGI respectively compared to 0.71 for GENE76; Supplementary Figure 12). However this difference was not statistically significant (Supplementary Table 5). Opposed to these high indices, the clinical risk calculated using Adjuvant! Online only displayed a concordance index of 0.69.

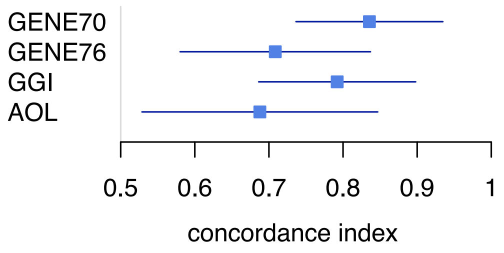


Supplementary Figure 12: Forest plots (and 95% CI) for the three gene signatures and the Adjuvant! Online classification showing the concordance indices considering TDM as survival endpoint.

| | p-value for difference in concordance indices | p-value for difference in hazard ratios |
|---|---|---|
| GENE70 vs GGI | 0.23 | 0.54 |
| GENE76 vs GENE70 | 0.065 | 0.25 |
| GENE76 vs GGI | 0.18 | 0.34 |

Supplementary Table 5: P-values of the Student t-test for the difference between concordance indices and hazard ratios considering DMFS as survival endpoint.

We next performed univariate and multivariate Cox analyses, which included the traditional clinico-pathological parameters, for each signature separately. The univariate hazard ratios (HR) were 2.77 (95% CI: 1.41-5.43, p= 3.1 x $10^{-3}$), 1.76 (95% CI: 0.92-3.34; p= 8.6 x $10^{-2}$) and 2.41 (95% CI: 1.29-4.5, p= 5.9 x $10^{-3}$) for the 70-gene signature, 76-gene signature and the GGI respectively. We additionally computed the HR for the clinical risk as defined by Adjuvant! Online that is equal to 1.8; 95% CI: 0.88- 3.7; p= 0.11). The $\log_2$ of these HR are illustrated in Supplementary Figure 13. Although the HR of the 70-gene signature and the GGI were higher than the HR of the 76-gene signature, the differences were not statistically significant (see Supplementary Table 5).

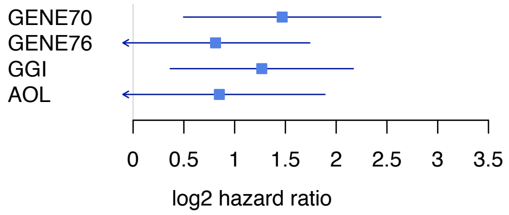


Supplementary Figure 13: Forest plots (and 95% CI) for the three gene signatures and the Adjuvant! Online classification showing the $\log_2$ hazard ratios considering TDM as survival endpoint.

Supplementary Figures 14, 15 and 16 illustrate the Kaplan-Meier estimates of TDM for the four groups of patients (two groups with concordant results in risk assessment and two with discordant results) for the different signatures two by two.

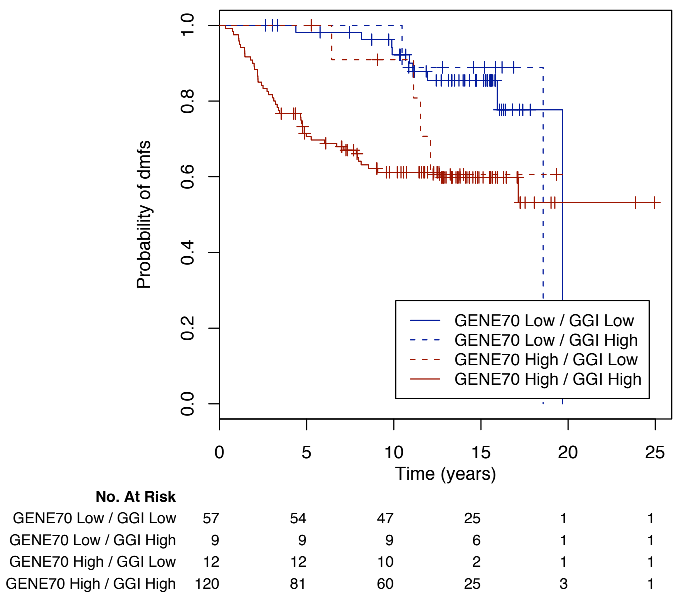


Supplementary Figure 14: Kaplan-Meier curves for time to distant metastasis for the 70-gene signature vs the genomic grade signature.

From the multivariate analyses (Supplementary Table 6), we can conclude that the three signatures added significant information to the traditional parameters and were the strongest predictive variables of DMFS, as reflected by their lowest p-values compared to the other variables. The additional information of these signatures over the clinical risk was also confirmed by the fact that the univariate HRs for the three signatures remained similar when adjusted for the clinical risk, with a HR of 2.8 (95% CI: 1.35-5.82, p=5.8 x $10^{-3}$), 1.55 (95% CI: 0.81-2.97, p=0.18) and 2.13 (95% CI: 1.12-4.02, p=2 x $10^{-2}$) for the 70-gene signature, 76-gene signature and the genomic grade signature respectively.

The Supplementary Table 7 reports the sensitivity and the specificity estimates at 3, 5, 10, 15 years and the full follow-up for all the classifiers.

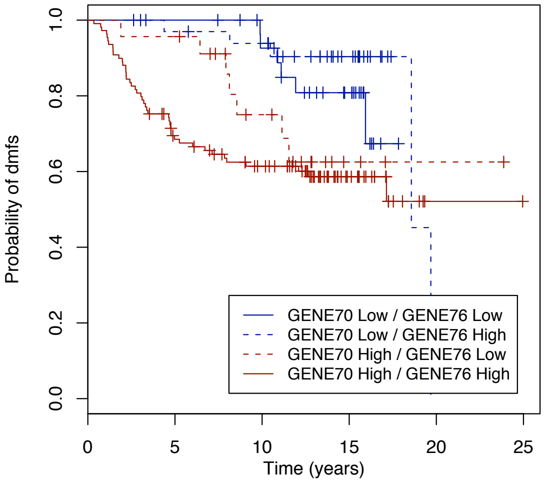


**No. At Risk**

| | | | | | | |
|---|---|---|---|---|---|---|
| GENE70 Low / GENE76 Low | 32 | 31 | 26 | 15 | 1 | 1 |
| GENE70 Low / GENE76 High | 34 | 33 | 31 | 16 | 1 | 1 |
| GENE70 High / GENE76 Low | 23 | 23 | 14 | 4 | 2 | 1 |
| GENE70 High / GENE76 High | 109 | 71 | 56 | 23 | 2 | 1 |

Supplementary Figure 15: Kaplan-Meier curves for time to distant metastasis for the 70-gene signature vs the 76-gene signature.

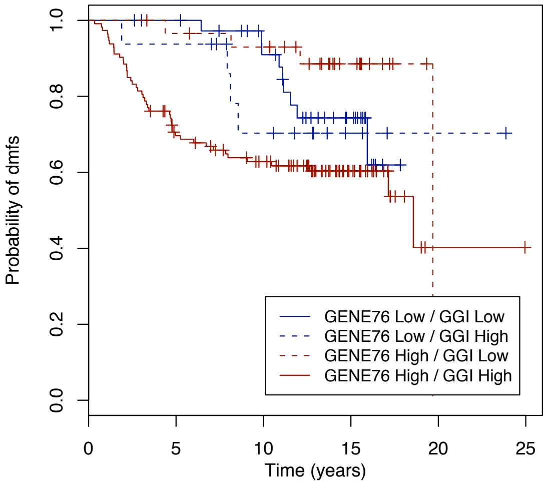


**No. At Risk**

| | | | | | | |
|---|---|---|---|---|---|---|
| GENE76 Low / GGI Low | 39 | 38 | 30 | 15 | 1 | 1 |
| GENE76 Low / GGI High | 16 | 16 | 10 | 4 | 2 | 1 |
| GENE76 High / GGI Low | 30 | 29 | 27 | 12 | 1 | 1 |
| GENE76 High / GGI High | 113 | 75 | 60 | 27 | 2 | 1 |

Supplementary Figure 16: Kaplan-Meier curves for time to distant metastasis for the 76-gene signature vs the genomic grade signature.

| | GENE70 | | GENE76 | | GGI | |
|---|---|---|---|---|---|---|
| | HR (95% CI) | p-value | HR (95% CI) | p-value | HR (95% CI) | p-value |
| Age ($\leq$ or $>$ 50years) | 1.33 (0.76-2.33) | 0.33 | 1.48 (0.85-2.59) | 0.17 | 1.47 (0.84-2.56) | 0.18 |
| Tumor size ($\leq$ or $>$ 2cm) | 1.52 (0.87-3.65) | 0.15 | 1.42 (0.81-2.48) | 0.22 | 1.43 (0.82-2.51) | 0.21 |
| ER status | 0.7 (0.37-1.33) | 0.28 | 0.54 (0.29-1.02) | 0.059 | 0.55 (0.35-1.24) | 0.19 |
| Grade | 0.54 (0.22-1.31) | 0.17 | 0.72 (0.29-1.78) | 0.48 | 0.46 (0.18-1.16) | 0.098 |
| Risk according to the gene signature | 3.08 (1.48-6.41) | $2.7 \times 10^{-3}$ | 1.9 (1.01-3.79) | $4.8 \times 10^{-2}$ | 2.93 (1.44-5.96) | $3 \times 10^{-3}$ |

Supplementary Table 6: Multivariate Cox analyses for the 70-gene signature (GENE70), the 76-gene signature (GENE76) and the genomic grade (GGI) risk classifications considering DMFS as survival endpoint.

| Classifier | Estimate | 3 years | 5 years | 10 years | 15 years | Full follow-up |
|---|---|---|---|---|---|---|
| GENE70 | Sensitivity | 1.00 | 0.97 | 0.92 | 0.86 | 0.82 |
| | Specificity | 0.38 | 0.40 | 0.42 | 0.41 | 0.40 |
| GENE76 | Sensitivity | 1.00 | 1.00 | 0.87 | 0.80 | 0.80 |
| | Specificity | 0.31 | 0.33 | 0.32 | 0.31 | 0.31 |
| GGI | SSensitivity | 1.00 | 0.97 | 0.90 | 0.80 | 0.78 |
| | Specificity | 0.39 | 0.42 | 0.43 | 0.41 | 0.41 |
| AOL | Sensitivity | 0.90 | 0.88 | 0.85 | 0.86 | 0.85 |
| | Specificity | 0.25 | 0.26 | 0.26 | 0.27 | 0.27 |

Supplementary Table 7: Sensitivity and specificity of the risk classifications computed by the 70-gene signature (GENE70), the 76-gene signature (GENE76), the genomic grade (GGI) and the Adjuvant! Online (AOL), considering DMFS as survival endpoint.

### 2.1.1 Combining GENE70, GENE76 and GGI in TBVDX

In order to assess the potential improvement in prognostication by combining the classifications computed from the three gene signatures, we applied the method described in Section 1.1.1.

The Supplementary Figure 17 sketches the survival curves for each category.

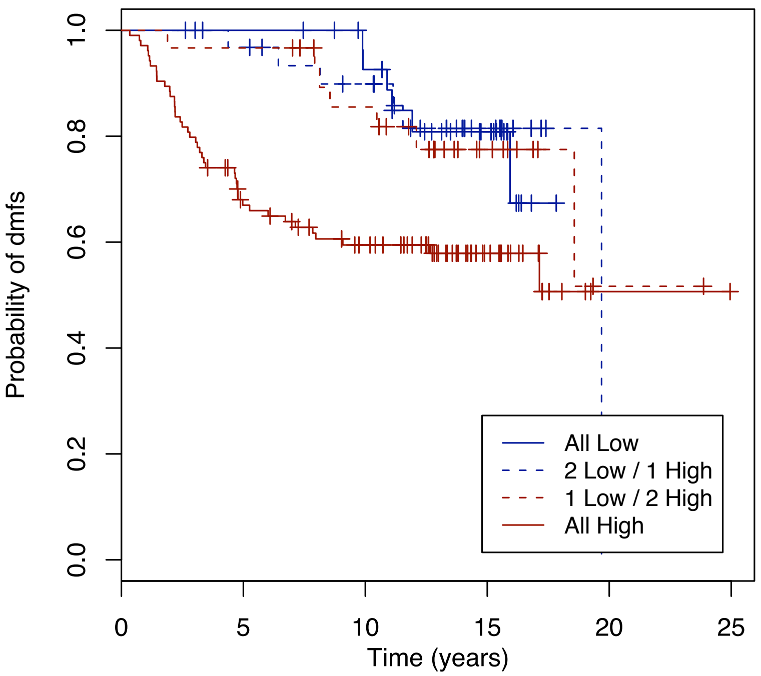


| No. At Risk | | | | | | |
|---|---|---|---|---|---|---|
| All Low | 32 | 31 | 26 | 15 | 1 | 1 |
| 2 Low / 1 High | 32 | 31 | 26 | 11 | 1 | 1 |
| 1 Low / 2 High | 30 | 30 | 24 | 10 | 2 | 1 |
| All High | 104 | 66 | 51 | 22 | 2 | 1 |

Supplementary Figure 17: Combination of the three gene signature classifications.

## 2.2 Time to Distant Metastasis (TDM)

To compare the prognostic ability of the three signatures considering TDM as survival endpoint, we first compared their concordance index, which is used to quantify the predictive ability of a survival model. Although the three indices were highly significant, the 70-gene and genomic grade signatures displayed a higher concordance index compared to the 76-gene signature (0.88 and 0.83 for GENE70 and GGI respectively compared to 0.78 for GENE76; Supplementary Figure 18). However this difference was not statistically significant (Supplementary Table 8). Opposed to these high indices, the clinical risk calculated using Adjuvant! Online only displayed a concordance index of 0.75.

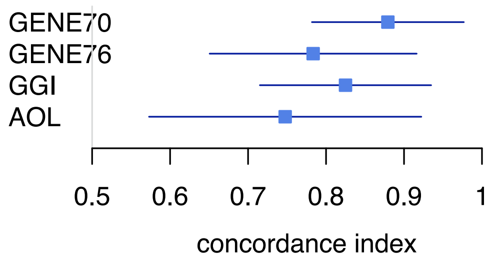


Supplementary Figure 18: Forest plots (and 95% CI) for the three gene signatures and the Adjuvant! Online classification showing the concordance indices considering TDM as survival endpoint.

| | p-value for difference in concordance indices | p-value for difference in hazard ratios |
|---|---|---|
| GENE70 vs GGI | 0.23 | 0.52 |
| GENE76 vs GENE70 | 0.14 | 0.45 |
| GENE76 vs GGI | 0.52 | 0.79 |

Supplementary Table 8: P-values of the Student t-test for the difference between concordance indices and hazard ratios considering TDM as survival endpoint.

We next performed univariate and multivariate Cox analyses, which included the traditional clinico-pathological parameters, for each signature separately. The univariate hazard ratios (HR) were 3.6 (95% CI: 1.59-8.16, p= 2.1 x $10^{-3}$), 2.63 (95% CI: 1.17-5.9; p= 1.9 x $10^{-2}$) and 2.93 (95% CI: 1.41-6.1, p= 4.1 x $10^{-3}$) for the 70-gene signature, 76-gene signature and the GGI respectively. We additionally computed the HR for the clinical risk as defined by Adjuvant! Online that is equal to 2.24; 95% CI: 0.94- 5.31; p= 6.8 x $10^{-2}$). The $\log_2$ of these HR are illustrated in Supplementary Figure 19. Although the HR of the 70-gene signature and the GGI were higher than the HR of the 76-gene signature, the differences were not statistically significant (see Supplementary Table 8).

Supplementary Figures 20, 21 and 22 illustrate the Kaplan-Meier estimates of TDM for the four groups of patients (two groups with concordant results in risk assessment and two with discordant results) for the different signatures two by two.

From the multivariate analyses (Supplementary Table 9), we can conclude that the three signatures added significant information to the traditional parameters and were the strongest

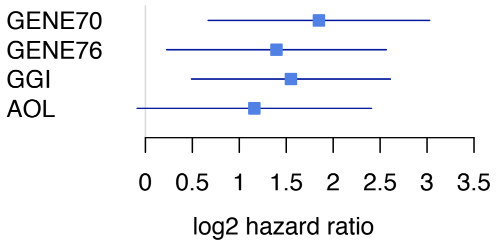


Supplementary Figure 19: Forest plots (and 95% CI) for the three gene signatures and the Adjuvant! Online classification showing the $\log_2$ hazard ratios considering TDM as survival endpoint.

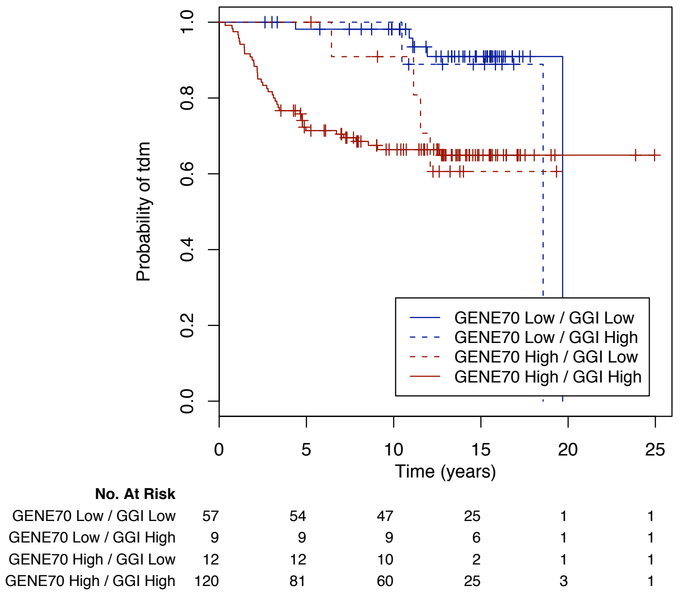


Supplementary Figure 20: Kaplan-Meier curves for time to distant metastasis for the 70-gene signature vs the genomic grade signature.

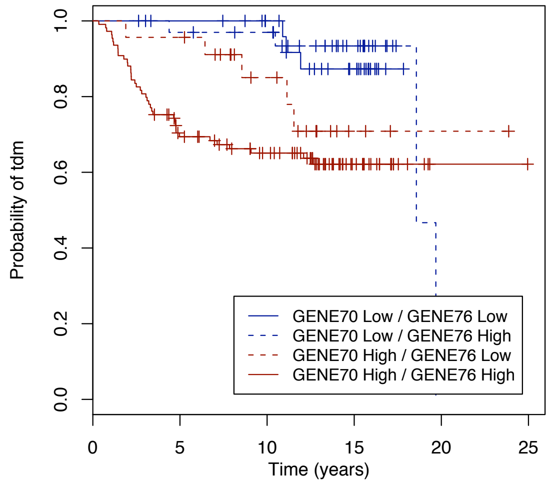


**No. At Risk**

| | | | | | | |
|---|---|---|---|---|---|---|
| GENE70 Low / GENE76 Low | 32 | 31 | 26 | 15 | 1 | 1 |
| GENE70 Low / GENE76 High | 34 | 33 | 31 | 16 | 1 | 1 |
| GENE70 High / GENE76 Low | 23 | 23 | 14 | 4 | 2 | 1 |
| GENE70 High / GENE76 High | 109 | 71 | 56 | 23 | 2 | 1 |

Supplementary Figure 21: Kaplan-Meier curves for time to distant metastasis for the 70-gene signature vs the 76-gene signature.

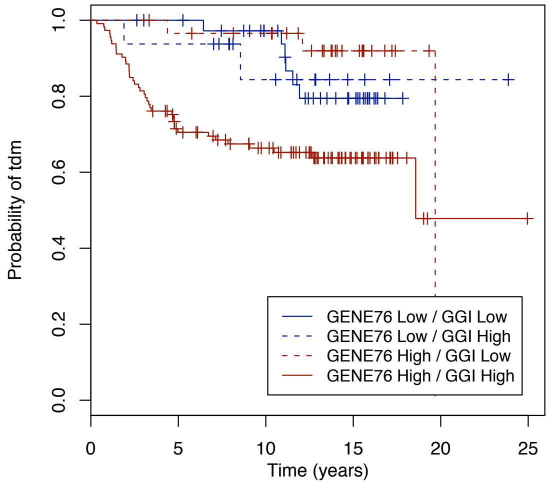


**No. At Risk**

| | | | | | | |
|---|---|---|---|---|---|---|
| GENE76 Low / GGI Low | 39 | 38 | 30 | 15 | 1 | 1 |
| GENE76 Low / GGI High | 16 | 16 | 10 | 4 | 2 | 1 |
| GENE76 High / GGI Low | 30 | 29 | 27 | 12 | 1 | 1 |
| GENE76 High / GGI High | 113 | 75 | 60 | 27 | 2 | 1 |

Supplementary Figure 22: Kaplan-Meier curves for time to distant metastasis for the 76-gene signature vs the genomic grade signature.

predictive variables of TDM, as reflected by their lowest p-values compared to the other variables. The additional information of these signatures over the clinical risk was also confirmed by the fact that the univariate HRs for the three signatures remained similar when adjusted for the clinical risk, with a HR of 3.69 (95% CI: 1.51-9.02, p=4.1 x $10^{-3}$), 2.32 (95% CI: 1.03-5.25, p=4.3 x $10^{-2}$) and 2.49 (95% CI: 1.19-5.23, p=1.6 x $10^{-2}$) for the 70-gene signature, 76-gene signature and the genomic grade signature respectively.

| | GENE70 | | GENE76 | | GGI | |
|---|---|---|---|---|---|---|
| | HR (95% CI) | p-value | HR (95% CI) | p-value | HR (95% CI) | p-value |
| Age ($\leq$ or $>$ 50years) | 1.25 (0.67-2.35) | 0.48 | 1.46 (0.79-2.73) | 0.23 | 1.39 (0.75-2.59) | 0.3 |
| Tumor size ($\leq$ or $>$ 2cm) | 1.66 (0.89-3.11) | 0.11 | 1.54 (0.83-2.86) | 0.17 | 1.56 (0.83-2.91) | 0.17 |
| ER status | 0.62 (0.41-1.63) | 0.57 | 0.62 (0.31-1.23) | 0.059 | 0.76 (0.39-1.5) | 0.43 |
| Grade | 0.74 (0.28-2) | 0.56 | 1.03 (0.372-2.86) | 0.95 | 0.64 (0.23-1.81) | 0.4 |
| Risk according to the gene signature | 3.79 (1.57-9.14) | 3 x $10^{-3}$ | 2.82 (1.23-6.46) | 1.4 x $10^{-2}$ | 3.22 (1.41-7.37) | 5.6 x $10^{-3}$ |

Supplementary Table 9: Multivariate Cox analyses for the 70-gene signature (GENE70), the 76-gene signature (GENE76) and the genomic grade (GGI) risk classifications considering TDM as survival endpoint.

The Supplementary Table 10 reports the sensitivity and the specificity estimates at 3, 5, 10, 15 years and the full follow-up for all the classifiers.

| Classifier | Estimate | 3 years | 5 years | 10 years | 15 years | Full follow-up |
|---|---|---|---|---|---|---|
| GENE70 | Sensitivity | 1.00 | 0.97 | 0.97 | 0.89 | 0.86 |
| | Specificity | 0.38 | 0.40 | 0.41 | 0.41 | 0.40 |
| GENE76 | Sensitivity | 1.00 | 1.00 | 0.95 | 0.85 | 0.86 |
| | Specificity | 0.31 | 0.33 | 0.33 | 0.32 | 0.32 |
| GGI | Sensitivity | 1.00 | 0.97 | 0.95 | 0.83 | 0.82 |
| | Specificity | 0.39 | 0.42 | 0.43 | 0.41 | 0.41 |
| AOL | Sensitivity | 0.90 | 0.91 | 0.90 | 0.89 | 0.88 |
| | Specificity | 0.25 | 0.26 | 0.27 | 0.28 | 0.27 |

Supplementary Table 10: Sensitivity and specificity of the risk classifications computed by the 70-gene signature (GENE70), the 76-gene signature (GENE76), the genomic grade (GGI) and the Adjuvant! Online (AOL), considering TDM as survival endpoint.

## 2.3 Overall Survival (OS)

To compare the prognostic ability of the three signatures considering OS as survival endpoint, we first compared their concordance index, which is used to quantify the predictive ability of a survival model. Although the three indices were highly significant, the 70-gene and genomic grade signatures displayed a higher concordance index compared to the 76-gene signature (0.86 and 0.83 for GENE70 and GGI respectively compared to 0.76 for GENE76; Supplementary Figure 23). However this difference was not statistically significant (Supplementary Table 11). Opposed to these high indices, the clinical risk calculated using Adjuvant! Online only displayed a concordance index of 0.67.

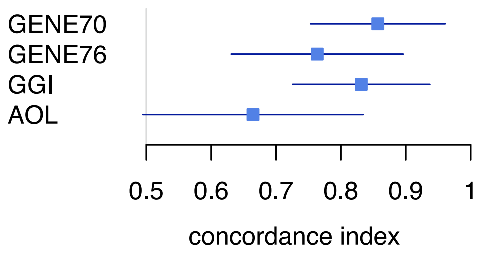


Supplementary Figure 23: Forest plots (and 95% CI) for the three gene signatures and the Adjuvant! Online classification showing the concordance indices considering OS as survival endpoint.

| | p-value for difference in concordance indices | p-value for difference in hazard ratios |
|---|---|---|
| GENE70 vs GGI | 0.49 | 0.42 |
| GENE76 vs GENE70 | 0.19 | 0.15 |
| GENE76 vs GGI | 0.29 | 0.26 |

Supplementary Table 11: P-values of the Student t-test for the difference between concordance indices and hazard ratios considering OS as survival endpoint.

We next performed univariate and multivariate Cox analyses, which included the traditional clinico-pathological parameters, for each signature separately. The univariate hazard ratios (HR) were 4.11 (95% CI: 1.82-9.27, p= 6.6 x $10^{-4}$), 2.14 (95% CI: 1.03-4.42; p= 4 x $10^{-2}$) and 3.3 (95% CI: 1.6-6.81, p= 1.2 x $10^{-3}$) for the 70-gene signature, 76-gene signature and the GGI respectively. We additionally computed the HR for the clinical risk as defined by Adjuvant! Online that is equal to 1.85; 95% CI: 0.86- 3.96; p= 0.12). The $\log_2$ of these HR are illustrated in Supplementary Figure 24. Although the HR of the 70-gene signature and the GGI were higher than the HR of the 76-gene signature, the differences were not statistically significant (see Supplementary Table 11).

Supplementary Figures 25, 26 and 27 illustrate the Kaplan-Meier estimates of OS for the four groups of patients (two groups with concordant results in risk assessment and two with discordant results) for the different signatures two by two.

From the multivariate analyses (Supplementary Table 12), we can conclude that the three signatures added significant information to the traditional parameters and were the strongest

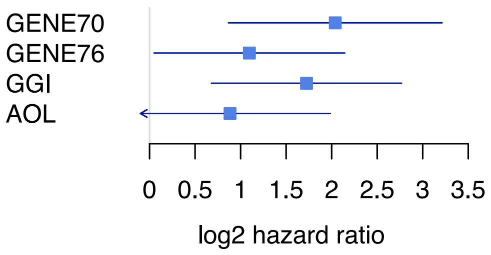


Supplementary Figure 24: Forest plots (and 95% CI) for the three gene signatures and the Adjuvant! Online classification showing the $\log_2$ hazard ratios considering OS as survival endpoint.

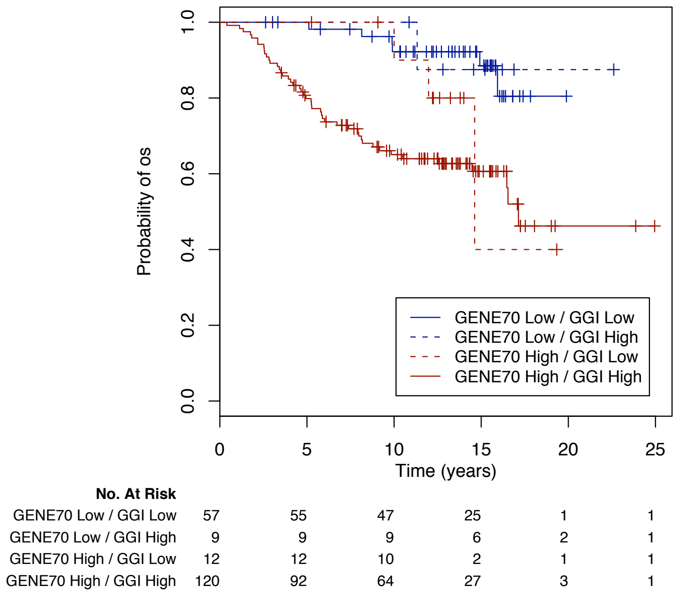


Supplementary Figure 25: Kaplan-Meier curves for time to distant metastasis for the 70-gene signature vs the genomic grade signature.

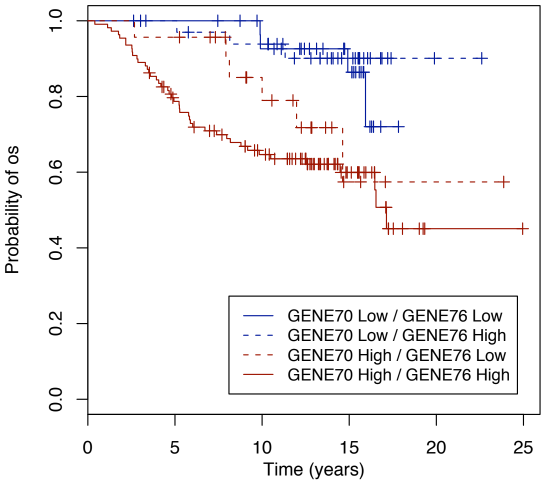


| **No. At Risk** | | | | | | |
|---|---|---|---|---|---|---|
| GENE70 Low / GENE76 Low | 32 | 31 | 26 | 15 | 1 | 1 |
| GENE70 Low / GENE76 High | 34 | 34 | 31 | 16 | 2 | 1 |
| GENE70 High / GENE76 Low | 23 | 23 | 14 | 4 | 2 | 1 |
| GENE70 High / GENE76 High | 109 | 82 | 60 | 25 | 2 | 1 |

Supplementary Figure 26: Kaplan-Meier curves for time to distant metastasis for the 70-gene signature vs the 76-gene signature.

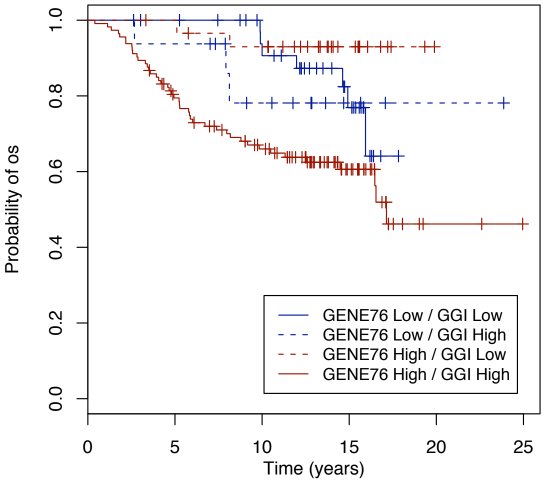


| **No. At Risk** | | | | | | |
|---|---|---|---|---|---|---|
| GENE76 Low / GGI Low | 39 | 38 | 30 | 15 | 1 | 1 |
| GENE76 Low / GGI High | 16 | 16 | 10 | 4 | 2 | 1 |
| GENE76 High / GGI Low | 30 | 30 | 27 | 12 | 1 | 1 |
| GENE76 High / GGI High | 113 | 86 | 64 | 29 | 3 | 1 |

Supplementary Figure 27: Kaplan-Meier curves for time to distant metastasis for the 76-gene signature vs the genomic grade signature.

predictive variables of OS, as reflected by their lowest p-values compared to the other variables. The additional information of these signatures over the clinical risk was also confirmed by the fact that the univariate HRs for the three signatures remained similar when adjusted for the clinical risk, with a HR of 3.91 (95% CI: 1.66-9.17, p=1.8 x $10^{-3}$), 1.93 (95% CI: 0.93-4, p=7.8 x $10^{-2}$) and 3 (95% CI: 1.44-6.28, p=3.5 x $10^{-3}$) for the 70-gene signature, 76-gene signature and the genomic grade signature respectively.

| | GENE70 | | GENE76 | | GGI | |
|---|---|---|---|---|---|---|
| | HR (95% CI) | p-value | HR (95% CI) | p-value | HR (95% CI) | p-value |
| Age ($\leq$ or $>$ 50years) | 1.35 (0.74-2.48) | 0.33 | 1.56 (0.86-2.83) | 0.14 | 1.6 (0.88-2.91) | 0.12 |
| Tumor size ($\leq$ or $>$ 2cm) | 1.44 (0.8-2.59) | 0.22 | 1.35 (0.75-2.44) | 0.32 | 1.36 (0.75-2.48) | 0.31 |
| ER status | 0.67 (0.35-1.29) | 0.23 | 0.49 (0.26-0.94) | 0.032 | 0.63 (0.33-1.2) | 0.16 |
| Grade | 0.52 (0.21-1.3) | 0.16 | 0.74 (0.29-1.88) | 0.52 | 0.42 (0.17-1.09) | 0.074 |
| Risk according to the gene signature | 4.59 (1.93-10.91) | 5.7 x $10^{-4}$ | 2.37 (1.12-5.01) | 2.4 x $10^{-2}$ | 4.29 (1.91-9.63) | 4.2 x $10^{-4}$ |

Supplementary Table 12: Multivariate Cox analyses for the 70-gene signature (GENE70), the 76-gene signature (GENE76) and the genomic grade (GGI) risk classifications considering OS as survival endpoint.

The Supplementary Table 13 reports the sensitivity and the specificity estimates at 3, 5, 10, 15 years and the full follow-up for all the classifiers.

| Classifier | Estimate | 3 years | 5 years | 10 years | 15 years | Full follow-up |
|---|---|---|---|---|---|---|
| GENE70 | Sensitivity | 1.00 | 1.00 | 0.91 | 0.88 | 0.87 |
| | Specificity | 0.36 | 0.38 | 0.41 | 0.41 | 0.42 |
| GENE76 | Sensitivity | 1.00 | 1.00 | 0.88 | 0.84 | 0.83 |
| | Specificity | 0.29 | 0.31 | 0.32 | 0.32 | 0.32 |
| GGI | Sensitivity | 1.00 | 1.00 | 0.88 | 0.84 | 0.83 |
| | Specificity | 0.37 | 0.40 | 0.42 | 0.42 | 0.42 |
| AOL | Sensitivity | 0.91 | 0.86 | 0.84 | 0.84 | 0.85 |
| | Specificity | 0.24 | 0.25 | 0.25 | 0.26 | 0.27 |

Supplementary Table 13: Sensitivity and specificity of the risk classifications computed by the 70-gene signature (GENE70), the 76-gene signature (GENE76), the genomic grade (GGI) and the Adjuvant! Online (AOL), considering OS as survival endpoint.
